# MiR-182-5p: A Novel Biomarker in the Treatment of Depression in CSDS-Induced Mice

**DOI:** 10.1093/ijnp/pyad064

**Published:** 2023-12-01

**Authors:** Ya-Bin Zheng, Xiao-Ming Sheng, Xiang Jin, Wei Guan

**Affiliations:** Department of Neurology, The second Hospital of Nanjing, Nanjing University of Chinese Medicine, Nanjing, Jiangsu, China; Department of Trauma Center, Affiliated Hospital of Nantong University, Nantong, Jiangsu, China; Department of Pharmacy, The Second People’s Hospital of Nantong, Nantong, Jiangsu, China; Department of Pharmacology, Pharmacy College, Nantong University, Nantong, Jiangsu, China

**Keywords:** Mechanism, MiR-182-5p, depression, chronic social defeat stress, CREB, neuronal neurogenesis

## Abstract

**Background:**

Depression is a neuropsychiatric disease with a high disability rate and mainly caused by the chronic stress or genetic factors. There is increasing evidence that microRNAs (miRNAs) play a critical role in the pathogenesis of depression. However, the underlying molecular mechanism for the pathophysiology of depression of miRNA remains entirely unclear so far.

**Methods:**

We first established a chronic social defeat stress (CSDS) mice model of depression, and depression-like behaviors of mice were evaluated by a series of behavioral tests. Next, we detected several abundantly expressive miRNAs suggested in previous reports to be involved in depression and found miR-182-5p was selected as a candidate for analysis in the hippocampus. Then western blotting and immunofluorescence were used together to examine whether adeno-associated virus (AAV)-siR-182-5p treatment alleviated chronic stress–induced decrease in hippocampal Akt/GSK3β/cAMP-response element binding protein (CREB) signaling pathway and increase in neurogenesis impairment and neuroinflammation. Furthermore, CREB inhibitor was adopted to examine if blockade of Akt/GSK3β/CREB signaling pathway abolished the antidepressant actions of AAV-siR-182-5p in mice.

**Results:**

Knockdown of miR-182-5p alleviated depression-like behaviors and impaired neurogenesis of CSDS-induced mice. Intriguingly, the usage of agomiR-182-5p produced significant increases in immobility times and aggravated neuronal neurogenesis damage of mice. More importantly, it suggested that 666-15 blocked the reversal effects of AAV-siR-182-5p on the CSDS-induced depressive-like behaviors in behavioral testing and neuronal neurogenesis within hippocampus of mice.

**Conclusions:**

These findings indicated that hippocampal miR-182-5p/Akt/GSK3β/CREB signaling pathway participated in the pathogenesis of depression, and it might give more opportunities for new drug developments based on the miRNA target in the clinic.

## Graphical Abstract

**Graphical Abstract:**
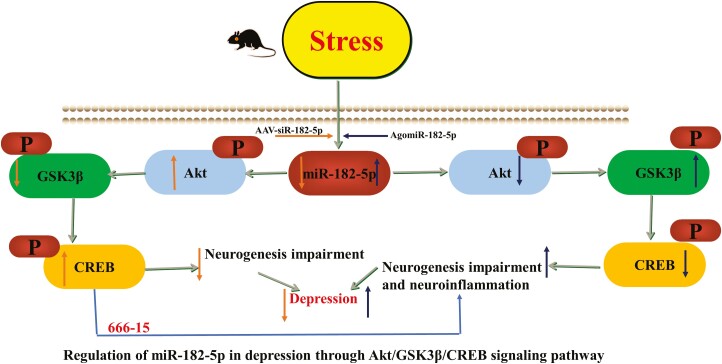

**Significance Statement**

Depression is one of the most severe psychiatric disorders that threatens both the physical and psychological health issues of the global population. microRNAs (miRNAs) are an important epigenetic process, and their abnormal expression may be involved in the pathogenesis of human diseases, including depression. In this study, we demonstrated that AAV-siR-182-5p treatment alleviated chronic stress–induced decrease in the hippocampal miR-182-5p/Akt/GSK3β/CREB signaling pathway and the increase in neurogenesis impairment and neuroinflammation. It was interesting to note that intranasal administration of AgomiR-182-5p not only induced notable depressive-like behaviors but also significantly decreased Akt/GSK3β/CREB signaling cascade. Considering the aggravated neurogenesis impairment and neuroinflammation in mice, we may for the first time develop novel nose drops based on the miRNA target in the clinic to relieve depression. These findings further prove that miR-182-5p could be a novel antidepressant target.

## INTRODUCTION

Depression is one of the most common diseases of neuroscience associated with cognitive impairment and a major cause of death and disability (Fox and Lobo, 2019). It is startling to realize that over 120 million patients do not adequately respond to antidepressant treatments, and casually increasing the dose of medicine often leads to significant side effects (Blumberg et al., 2020). Thus, it is necessary to research new therapeutic biomarkers of depression in the clinic treatment because they could be used as useful targets for the development of new drugs. However, the underlying molecular mechanisms and pathogenesis of depression remain unclear.

A growing body of research shows that depression is an effect of complex genetic, epigenetic, and environmental interactions, and people have recently paid more attention to the epigenetic factors (Kwong et al., 2019; Lin and Tsai, 2019). MicroRNAs (miRNAs) are important to the epigenetic process, and their abnormal expression may be involved in the pathogenesis of human diseases, including depression (Shi et al., 2021). miRNAs are a new class of small and single-stranded noncoding RNA molecules of approximately 20–22 nucleotides in length, which inhibits the expression of the target gene with a complementary seed that matches the 3' untranslated region (3'UTR) position. Reported evidence has demonstrated that miRNAs play precise roles in the expression of coding genes, neurogenesis, and neural plasticity (O’Connor et al., 2013; Dwivedi, 2014). Furthermore, several miRNAs such as miR-34 and miR-146a-5p have been reported to be associated with the complicated pathogenesis of depression (Yi et al., 2020; Fan et al., 2022).

The results we went on the basis of CSDS mice model, which was widely used to simulate some rodent models of depression in vivo, indicated that depression-like behaviors were accompanied with the decreased level of cAMP-response element binding protein (CREB) and neuronal cell damage (Jia et al., 2021; Wang et al., 2021). CREB, a key regulator of neuronal plasticity, plays an important role in synaptogenesis and long-term potentiation and thus is regarded as a key nucleoprotein related to depression and antidepressant treatments (Blendy, 2006). Glycogen synthase kinase 3β (GSK3β) has been shown to be relevant in the pathophysiology of mood disorders; GSK3β inhibitors may reduce neuroinflammation and are essential for strengthening CREB activity (Pláteník et al., 2014).

Furthermore, serine-threonine protein kinase (Akt), an upstream regulator of GSK3β, could facilitate GSK3β phosphorylation to inhibit GSK3β activity and thus has been implicated in modulating depression and other neuropsychopathies (Wang et al., 2021). However, the underlying mechanism through which downregulation of miR-182-5p counteracts stress-induced aberrant hippocampal neurogenesis via Akt/GSK3β/CREB signaling remains elusive.

miR-182-5p is a member of the miR-183 family located on chromosome 7q31-34 and described as an oncogenic miRNA due to its capacity to enhance cancer cell proliferation and survival (Cao et al., 2018). Although roles of miR-182-5p are well known in cancer, little is known about its function in the CNS under normal and pathophysiological conditions. In the present study, expression levels of 7 miRNAs previously reported to be depression-associated miRNAs (let-7a, miR-182-5p, miR-27a, miR-26a-3p, miR-323a, miR-139-5p, miR-199a-5p) were measured using real-time PCR (Table 1). It was found that the expression level of miR-182-5p was highest in the hippocampus of mice exposed to the CSDS model. In addition, the knockdown of miR-182-5p rescued depression-like phenotypes, increasing the Akt/GSK3β/CREB signaling pathway and neurogenesis in the hippocampus of CSDS-induced mice. Therefore, the miR-182-5p has the potential to emerge as a valuable biomarker in brain tissue, help diagnose disease, and predict responses to therapeutic interventions.

**Table 1.** Previously reported major depressive disorder–related microRNAs in animal or human samples

miRNA name	Source	Sample	Change	Authors
hsa-miR-182-5p	Patients with depression	Serum	Up	(Li et al., 2013)
hsa-let-7a	MDD patients	Blood	Down	(Maffioletti et al., 2016)
miR-27a	Depressed patients and mice	Serum	Down	(Li et al., 2021)
miR-26a-3p	CUMS-induced rats	Hippocampus	Down	Li et al., (2021)
miR-323	MDD patients	Anterior cingulate cortex	Up	(Fiori et al., 2021)
has-miR-139-5p	MDD patients	Blood	Up	(Wei et al., 2020)
miR-199a-5p	MDD patients	Cerebrospinal fluids and serums	Up	(Liu et al., 2021)

## MATERIALS AND METHODS

### Materials

miR-182-5p–related reagents were obtained from Beyotime Biotechnology Co., Ltd (Shanghai, China). We bought adeno-associated virus (AAV)-siR-182-5p and its negative control, AAV-Scrambled from Genechem Co., Ltd (Shanghai, China). 666-15 was purchased from Sigma-Aldrich (5383410001, St. Louis, MO). 666-15 is a potent CREB inhibitor and has been shown to possess efficacious anti-breast cancer activity without toxicity in vivo (Xie et al., 2017; Peng et al., 2022), which is dissolved in 1% Dimethyl sulfoxide (DMSO) in normal saline (vehicle) and i.p. injected at a dose of 10 mg/kg (Kaur et al., 2022).

### Animals

Adult male C57BL6/J mice (23–25 g, between 7 and 8 weeks old) were obtained from the Experimental Animal Centre of Medical College at the Nantong University (Nantong, China), and male retired CD1 mice (50–55 g, 8–9 months) were bought from Beijing Vital River Laboratories (Beijing, China). Initially, all mice were housed in plastic cases (5 per cage) and kept on a 12-hour-light/-dark cycle (lights on 7:30 am–6:30 pm). Ambient temperature was maintained at 24°C ± 1°C. Mice had ad libitum access to food and water at any time. Before the experiments, mice were acclimatized for 7 days. All the animal protocols and procedures were approved by the Institutional Animal Ethical Committee of Nantong University (approval no., 20171220-005) and conducted according to National Institutes of Health guidelines (Guan et al., 2021).

### Chronic Social Defeat Stress (CSDS)

The CSDS paradigm was conducted as previously described, with minor modifications (Kosuge et al., 2021; Shen et al., 2022). In the beginning of the study, aggressive CD1 mice were habituated on the right side of the cage (28 × 45 × 20 cm) divided using plastic glass separators for 30 minutes. C57BL/6J mice were exposed to different aggressive CD1 mice for 10 minutes each day for 10 consecutive days. After each stress exposure, the defeated mice were returned to another side of the cage as an aggressor mouse separated via a transparent plastic glass plate and were subjected to continuous psychological stress, such as visual stimuli, olfactory, and threatening auditory, for the remaining 24 hours. The control mice were separately grouped in pairs in the same cage and also exposed to 10 different C57BL/6 J mice for 10 consecutive days.

The social interaction test was used to assess anxiety-related and social avoidance behavior in rodents (Kaidanovich-Beilin et al., 2011). First, mice were placed in a clean and quiet room at near room temperature for 2 hours before the test was initiated. After habituation, the test C57BL/6J mice were individually exposed to 2 testing trials: “target absent” trial and “target present” trial. The mouse was transferred to an open field chamber (50 × 50 × 45 cm height), where a male CD-1 mouse was absent or present in the mesh cage at one end. A small, round, wire-mesh cage was placed in the interaction zone (14 × 26 cm). Each mouse was allowed to freely explore the environment for 5 minutes, and its behavior was monitored by a digital camera mounted above the arena (Ethovision, Noldus, the Netherlands). After each test session, feces and urine were removed and the arena cleaned with a 20% ethanol solution (Reguilón et al., 2022). The interaction ratio is a ratio of calculated time spent in the interaction zone with a CD-1 to the time spent in the interaction zone without a CD-1. In general, a mouse whose interaction ratio was <1 was regarded as a susceptible mouse, and a mouse whose interaction ratio was >1 was classified as resilient.

### Forced Swim Test (FST)

FST was performed using a transparent glass cylinder (60-cm height × 30-cm diameter) filled with 30 cm water (23°C ± 2°C). Mice were floated in the water for a total of 6 minutes. The total immobility time was recorded for the last 4 minutes by an investigator blinded to the study. The water in the cylinder was replaced at the end of each trial.

### Tail Suspension Test (TST)

The TST was performed as previously described with minor modifications (Shi et al., 2022). During the experiment, each mouse was individually suspended for 6 minutes on an apparatus by using medical tape placed 1 cm from the tip of the tail. The immobile latency for the first 2 minutes and time spent immobile were automatically recorded for the rest of the 4 minutes by XinRuan TST system (XR-XX203, Shanghai, China).

### Sucrose Preference Test (SPT)

The SPT was carried out to assess depression behaviors in the offspring, as previously reported (Liu et al., 2018; Qin et al., 2021). From the beginning, all mice were adapted to the experimentation room for 24 hours with 2 standard drinking bottles (1 containing 1% sucrose solution and the other with fresh water). Afterward, mice were deprived of food and water for 12 hours and then given preweighed bottles that contained 1% sucrose or water for 18 hours of testing (2 bottles needed to switch places every 6 hours). Eighteen hours later, the weights of the bottles were measured by the scale. The sucrose preference (%) index was calculated as SP (%) = sucrose water consumption (g)/[sucrose consumption (g) + water consumption (g)] × 100%.

### Real-Time Fluorescence Quantitative PCR (qRT-PCR)

qRT-PCR was performed with PowerUp SYBR Green Master mix (Thermo Fisher Scientific, Inc.) detection kit according to the manufacturer’s protocol. Total RNA was extracted from the tissue of hippocampus in mice using the RNA Extraction kits (Tiangen Biotech Co., Ltd., Beijing, China) and then reverse transcribed into cDNA. The process of miRNA reverse transcription occurred in an Applied Biosystems GeneAmp PCR System 9700 with SuperScript IV kits (Thermo Fisher Scientific, Inc.), and real-time PCR was carried out using the StepOne Plus System (Thermo Scientific, USA). qRT-PCR was performed using the following protocol: initial denaturation 95°C for 2 minutes, then 40 cycles at 95°C for 15 seconds, 60°C for 20 seconds, an the protocol ended with 72°C for 20 seconds. The relative fold change in expression of miRNA was determined using the 2-ΔΔCt method. U6 and GAPDH served as internal references (Gao and Zhang, 2021). The primer sequences used are listed in Table 2.

**Table 2.** Primer sequences

Name	Sequence (5’ to 3’)
GAPDH-S	CAAGCAACTGTCCCTGAG
GAPDH-A	TAGACAGAAGGTGGCACA
U6-S	ATTGGAACGATACAGAGAAG
U6-A	GGAACGCTTCACGAATTTG
miR-182-5p-S	AGCCGTTTGGCAATGGTAGAACTC
miR-182-5p-A	GTGCAGGGTCCGAGGT
miR-193a-5p-S	CAGTGCAGGGTCCGAGGT
miR-193a-5p-A	AACAATTGGGTCTTTGCGGGC
miR-142-5p-S	CATAAAGTAGAAAGCACTAC
miR-142-5p-A	GAACATGTCTGCGTATCTC
hsa-let-7a-S	CGCGCGCTATACAATCTACTGT
hsa-let-7a-A	AGTGCAGGGTCCGAGGTATT
miR-27a-S	GCGCGTTCACAGTGGCTAAG
miR-27a-A	AGTGCAGGGTCCGAGGTATT
miR-26a-3p-S	GCGCGCCTATTCTTGGTTACT
miR-26a-3p-A	AGTGCAGGGTCCGAGGTATT
miR-323-S	GCGCACATTACACGGTCG
miR-323-A	AGTGCAGGGTCCGAGGTATT
miR-139-5p-S	CGCGTCTACAGTGCACGTGT
miR-139-5p-A	AGTGCAGGGTCCGAGGTATT
miR-199a-5p-S	CGCGCCCAGTGTTCAGACTAC
miR-199a-5p-A	AGTGCAGGGTCCGAGGTATT

### Virus-Mediated Gene Transfer

The injection was performed as previously described with slight modification (Shi et al., 2022). Briefly, mice were anaesthetized with 0.5% sodium pentobarbital and secured via ear bars and incisor bar on a Kopf stereotaxic frame (Stoelting, USA). The microliter syringes of 5 μL were used to deliver the AAV to the target regions. The position of viral injections was slightly modified according to our previous study using the following coordinates: from the bregma, −2.45 mm; medial/lateral, ± 1.6 mm; and dorsal/ventral, + 1.8 mm. The purified AAV was infused bilaterally into the hippocampus regions at a rate of 0.2 μL/min (1 μL per side). The microliter syringes were maintained in the injection site for at least 5 minutes to prevent virus reverse flow. The incision of each mouse was sutured, and mice were carefully monitored postsurgery until fully recovered.

### Immunofluorescence

The mice from each group were anesthetized with 0.5% sodium pentobarbital (30 mg/kg) and pericardially perfused with 0.9% saline then fixed in 1000 mL 4% paraformaldehyde overnight. Brains were removed and placed in 4% paraformaldehyde for post-fixation (24 hours, 4°C). Brains were dehydrated in 30% sucrose (48 hours, 4°C), and then optimal cutting temperature compound was embedded. Next, the 25-μm-thick sections of the brains were cut. The slides were washed in phosphate buffered saline with 0.5% Triton X-100 (Beyotime, China) for 5–10 minutes, incubated in a blocking solution containing 3% bovine serum albumin (BSA) for 2 hours at room temperature, and the following primary antibody diluted in 3% BSA—doublecortin (1:300; Abcam; ab207175), GFAP (1:5000; Abcam; ab7260), and Iba-1 (1:100; Abcam; ab178847)—were incubated overnight at 4°C. Third, the Alexa Fluor 555 Goat Anti-Mouse IgG (1:800, Abcam; ab150114) or Alexa Fluor 488 Goat Anti-Mouse IgG (1:500; Abcam; ab150113) was added to incubate the sections for 2 hours at 37°C. Finally, the nuclei were then stained with 4'-6-diamidino-2-phenylindole (DAPI) for 10 minutes. Fluorescent images were captured with a fluorescence microscope (SP8; Leica, German) (Dang et al., 2022).

### Western Blotting Analysis

Total proteins were placed and mixed with a vortex oscillator for about 30 minutes in 100 μL RIPA lysis buffer (Beyotime Biotechnology, China) on ice, the extract was centrifuged, and the supernatant was collected in centrifuge tubes. Protein concentrations were determined with a BCA Protein Assay kit (Beyotime Biotechnology, China). Equivalent amounts of protein were loaded in sodium dodecyl sulfate-polyacrylamide gel electrophoresis gels and electrophoretically separated at a constant voltage of 100V for 2 hours. Subsequently, proteins were transferred to polyvinylidene difluoride membranes (Millipore, Billerica, MA, USA). The membranes were incubated in a block buffer (TBS-0.1% Tween 20 containing 5% skimmed milk) for 2 hours at room temperature. Then, the membranes were treated overnight at 4°C with the different antibodies: anti-beta Actin (1:1000; Abcam; ab8224), rabbit anti-AKT (phospho T308) (1:1000; Abcam; ab38449), rabbit anti-GSK3β (phospho S9) (1:5000; Abcam; ab107166), and rabbit anti-CREB (phospho S133) (1:5000; Abcam; ab32096). Next, membranes were incubated for an additional 2 hours with horseradish peroxidase–conjugated secondary antibody (1:1000; Cell Signaling; 7076S) at room temperature. The membrane was detected using enhanced chemiluminescence (Cell Signaling; 12630) reagent (Cai et al., 2021; Li et al., 2022).

### Statistical Analysis

The statistical analyses and bar graphs were conducted with the GraphPad Prism 6.0. The differences among treatment groups were analyzed using the SPSS software including 1-way or 2-way ANOVA as appropriate, by Tukey post hoc test or Bonferroni test for multiple comparisons. Results were considered significant when *P < *.05.

## RESULTS

### MiR-182-5p Increased Within the Hippocampal DG Region of CSDS Mice

**Figure 1.**
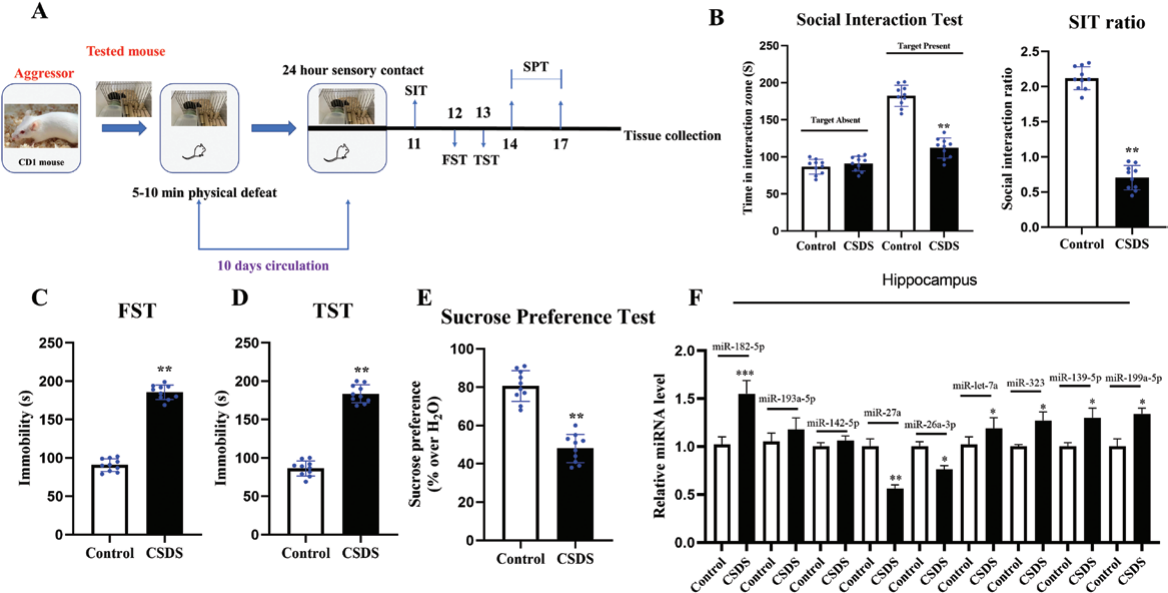
Chronic social defeat stress (CSDS) robustly induced depressive-like behaviors of normal mice. (A) Schematic timeline of CSDS and behavioral tests. (B–E) CSDS induced notable depressive-like actions of C57BL/6J mice in the FST, TST, SPT, and SIT (n = 10). (F) CSDS enhanced the level of miR-182-5p in the hippocampus of mice by qRT-PCR (n = 3). Data are shown as mean ± SEM. ^**^P < .01 compared with the control. Student *t* tests were employed for the comparisons between the 2 groups.

We first established the CSDS-induced mice to examine the effects of CSDS on miRNA expressions in hippocampus (Fig. 1A). The day after the end of CSDS, we carried out behavioral tests and observed that mice in the CSDS group had lower social interaction (t = 7.834 > t_0.01,18_ = 3.142, *P < *.01) in the social interaction test (SIT), significantly increased immobility in swimming times (t = 10.435 > t_0.01,18_ = 4.726, *P* < .01) and the TST (t = 8.914 > t_0.01,18_ = 4.022, *P* < .01), and less sucrose consumption in the SPT (t = 4.997 > t_0.01,18_ = 2.359, *P* < .01). The result proves the validity of the depression model and means notable depressive-like behaviors (Fig. 1B–E; n = 10). Next, according to previous research (Singh et al., 2016; Roser et al., 2018; Deng et al., 2020; Huang et al., 2021; Soto et al., 2022), we screened several abundantly expressive miRNAs being related to the depression in the hippocampus. Among the differentially expressed miRNAs, the level of miR-182-5p was significantly upregulated, whereas the expression of miR-27a and miR-26a-3p was downregulated in mice in the CSDS group compared with control mice (Fig. 1F; n = 3).

### Genetic Knockdown of miR-182-5p in Hippocampus Alleviated Depression-Like Behaviors in CSDS Mice

**Figure 2.**
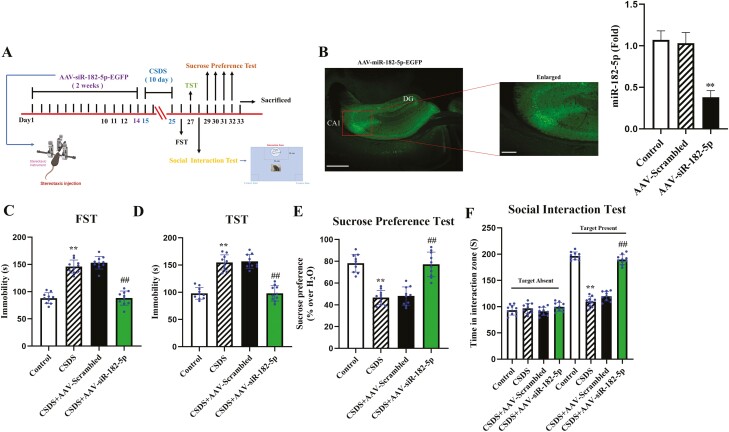
Knockdown of miR-182-5p within the hippocampus protected against CSDS in normal mice. (A) Experimental procedures for virus injection, CSDS, and behavioral testing. (B) Fluorescence image of AAV-siR-182-5p after 14 days stereotactic infusion that expressed in a fixed brain section. Scale bar = 400 µm. qRT-PCR was used to validate the knockdown efficiency of AAV-siR-182-5p (n = 3). (C–F) AAV-siR-182-5p infusion notably ameliorated the depression-like behaviors in CSDS-induced mice (n = 10). Data are presented as the means ± SEM. ^**^P < .01 compared with the control; ^##^*P < *.01 compared with the CSDS. One-way ANOVA + Tukey test was used for B. Two-way ANOVA followed by Bonferroni test was used for C–F.

In this experiment, to decide whether reversing the CSDS-induced high expression of miR-182-5p prevented depression-like behaviors, an AAV virus (AAV-siR-182-5p) was constructed to inhibit miR-182-5p expression within the hippocampal region of CSDS mice. AAV-siR-182-5p was stably expressed on the 14th day after stereotactic injection, and its knockdown efficacy was confirmed using qRT-PCR (Fig. 2A and B; n = 3). ANOVA showed that AAV-siR-182-5p treatment significantly relieved the CSDS-induced increasing effects on mouse immobility in the FST (interaction: F_2,36_ = 19.371, *P* < .01; CSDS: F_1,36_ = 39.246, *P* < .01; AAV: F_2,36_ = 24.863, *P < *.01) and TST (interaction: F_2,36_ = 20.449, *P* < .01; CSDS: F_1,36_ = 43.174, *P* < .01; AAV: F_2,36_ = 26.957, *P* < .01). Regarding the SPT, the ANOVA showed that AAV-siR-182-5p also prevented decreasing effects on sucrose consumption (interaction: F_2,36_ = 9.746, *P* < .01; CSDS: F_1,36_ = 19.352, *P* < .01; AAV: F_2,36_ = 14.582, *P* < .01) and social interaction (interaction: F_2,36_ = 38.927, *P* < .01; CSDS: F_1,36_ = 80.351, *P* < .01; AAV: F_2,36_ = 49.747, *P* < .01) in CSDS-induced mice (Fig. 2C–F; n = 10).

### Knockdown of miR-182-5p Prevented Decreasing Effects of CSDS on Neuronal Neurogenesis

**Figure 3.**
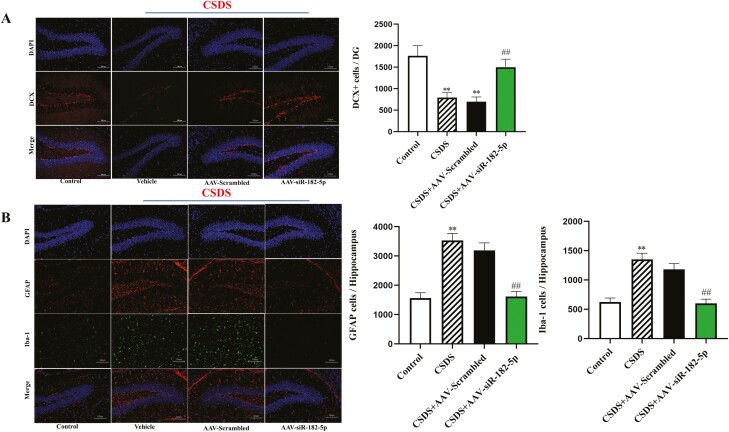
AAV-siR-182-5p treatment improved the hippocampal neurogenesis in the CSDS depression model, as revealed by immunofluorescence staining of DCX, GFAP, and Iba-1 cells in the hippocampus region (scale bar = 100 μm for representative images; n = 3). All data shown as means ± SEM. ^**^P < .01 compared with the control; ^##^*P < *.01 compared with the CSDS. The comparisons were made by (2-way ANOVA + Bonferroni test).

Depression is often characterized by impairments in adult neurogenesis (Kim and Park, 2021). To investigate a possible neuronal mechanism for the lower expression of miR-182-5p, we first examined changes in the amounts of doublecortin (DCX) cells by the immunofluorescence in the hippocampus among different groups. DCX, a widely accepted marker of newly generated granule cells, was detected in diverse human neurons, but it did not define immature neuron populations (Franjic et al., 2022). To further corroborate CSDS-induced neuroinflammation, we also measured ionized calcium-binding adaptor molecule 1 (Iba-1) and glial fibrillary acidic protein (GFAP) expression in the hippocampus of mice. ANOVA analysis from the results showed a lower number of DCX cells were observed in the CSDS group compared with the control group (Fig. 3A; n = 3), whereas a decreased number of DCX cells resulting from CSDS were completely reversed by AAV-siR-182-5p treatment [ANOVA: (interaction: F_2,16_ = 19.257, *P* < .01; CSDS: F_1,16_ = 38.416, *P* < .01; AAV: F_2,16_ = 26.837, *P* < .01)]. Our immunofluorescence results also indicated that CSDS significantly enhanced Iba-1 and GFAP expression in DG regions of the hippocampus. Interestingly, AAV-siR-182-5p treatment reversed the above CSDS-induced changes, including increased Iba-1 [ANOVA: (interaction: F_2,16_ = 21.853, *P* < .01; CSDS: F_1,16_ = 33.722, *P* < .01; AAV: F_2,16_ = 25.481, *P* < .01)] and GFAP [ANOVA: (interaction: F_2,16_ = 22.190, *P* < .01; CSDS: F_1,16_ = 42.373, *P* < .01; AAV: F_2,16 _= 31.069, *P* < .01)] expression (Fig. 3B; n = 3), suggesting the strong anti-inflammatory effects of AAV-siR-182-5p treatment.

### Downregulation of miR-182-5p Increased Akt/GSK3β/CREB Expression in Mice Exposed to CSDS

**Figure 4.**
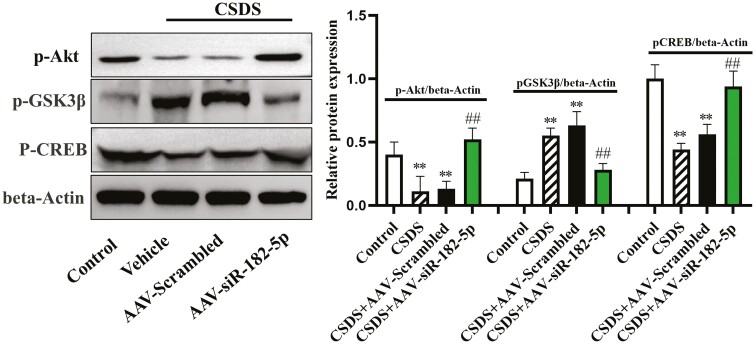
Genetic knockdown of miR-182-5p remarkably enhanced the hippocampal Akt/GSK3β/CREB signaling cascade in CSDS-stressed mice. Western-blot analysis showed that the promoting effects of AAV-siR-182-5p treatment on the expression levels of p-Akt and p-CREB were markedly higher than the CSDS group, whereas p-GSK3β expression was reduced. The results of analysis were expressed as means ± SEM, n = 3. ^**^P < .01 compared with control; ^##^*P < *.01 compared with CSDS. The comparisons were made by (2-way ANOVA + Bonferroni test).

To further prove the reliability of combination between miR-182-5p and AKT, a RIP assay was specially designed. The results showed that the levels of miR-182-5p and AKT were enriched in Argonaute 2 (AGO2) (supplementary Fig. 1) in the hippocampus of CSDS-induced mice. Afterwards, the expression levels of the hippocampal Akt/GSK3β/CREB signaling cascade were assessed by western blotting among all groups (n = 3). ANOVA showed that there was a significant difference regarding p-Akt, p-GSK3β, and p-CREB levels (Fig. 4); compared with the control group, the decreased p-Akt and p-CREB and increased p-GSK3β expression levels were seen in mice following 10 days of CSDS exposure. Moreover, the knockdown of miR-182-5p increased protein levels of p-Akt and p-CREB and decreased p-GSK3β expression in the hippocampus compared with CSDS [p-Akt: CSDS, F_(1,16)_ = 28.109, *P* < .01; AAV, F_(2,16)_ = 21.302, *P* < .01; interaction, F_(2,16)_ = 16.838, *P* < .01; p-GSK3β: CSDS, F_(1,16)_ = 32.174, *P* < .01; AAV, F_(2,16)_ = 22.487, *P* < .01; interaction, F_(2,16)_ = 17.366, *P* < .01; p-GSK3β: CSDS, F_(1,16)_ = 26.779, *P* < .01; AAV, F_(2,16)_ = 23.194, *P* < .01; interaction, F_(2,16)_ = 18.703, *P* < .01].

### Overexpression of miR-182-5p Induced Depression-Like Behaviors in Mice

**Figure 5.**
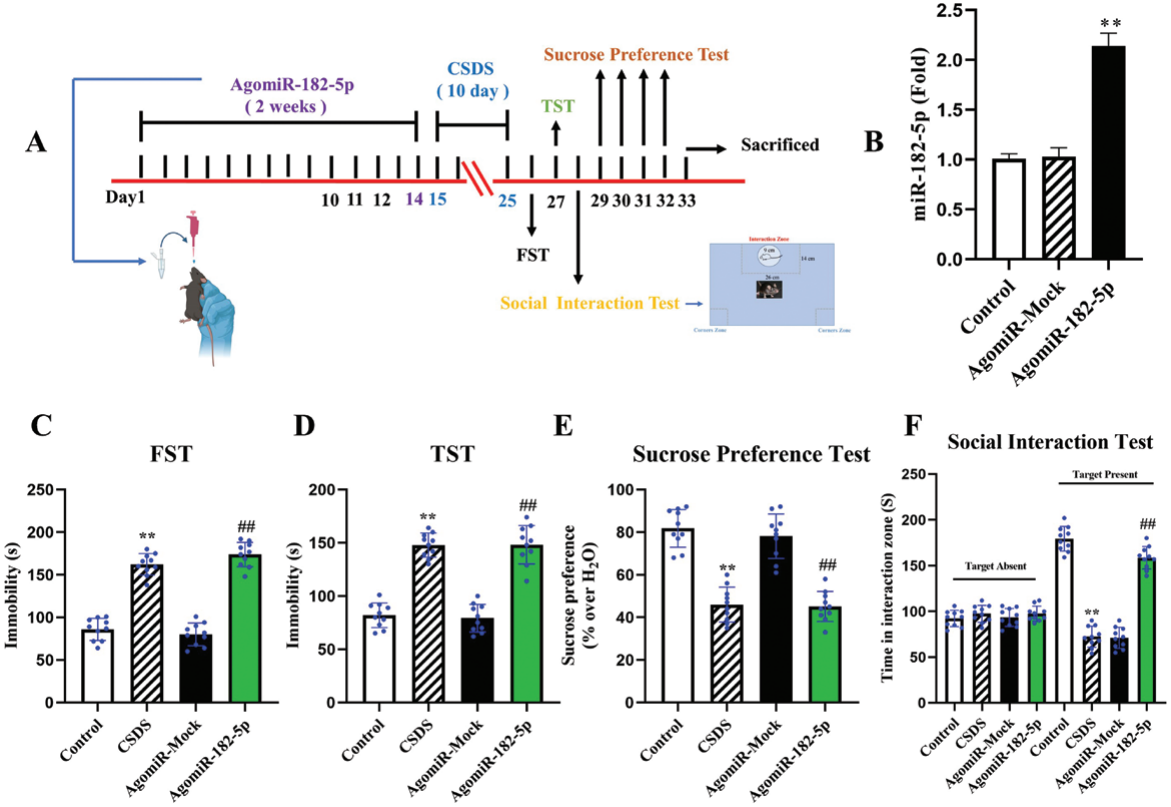
Overexpression of miR-182-5p exacerbated the depression-like behaviors in normal mice. (A) Experimental paradigm for intranasal injection, CSDS, and behavioral testing. (B) qRT-PCR was conducted to validate the efficiency of AgomiR-182-5p (n = 3). Intranasal administration of AgomiR-182-5p induced the depression-like behaviors in the FST (C), TST (D), SPT (E), and SIT (F) in normal mice (n = 10). Data are means ± SEM. ^**^P < .01 compared with the control; ^##^*P < *.01 compared with the AgomiR-Mock. One-way ANOVA + Tukey test was used for B–F.

To determine whether the upregulation of miR-182-5p was involved in producing notable depressive-like symptoms in mice, an agonist of miR-182-5p, AgomiR-182-5p, was i.p. injected into mice for 14 days, the high expressed efficiency of miR-182-5p was confirmed by qPCR, and a 115% increased level was calculated in hippocampus compared with the control group (*P* < .01; Fig. 5A and B; n = 3). Finally, the FST, TST, SPT, and SIT were conducted to examine depression-like behaviors (Fig. 5C–F; n = 10). The usage of agomiR-182-5p produced significant increases in the immobility of mice in the FST [ANOVA: F_(3,36)_ = 21.427, *P* < .01], TST [ANOVA: F_(3,36)_ = 23.844, *P* < .01], SPT [ANOVA: F_(3,36)_ = 10.430, *P* < .01], and SIT [ANOVA: F_(3,36)_ = 38.252, *P* < .01] compared with that observed in mice treated with a control injection (AgomiR-Mock).

### Overexpression of miR-182-5p Impaired Neuronal Neurogenesis and Suppressed Akt/GSK3β/CREB Signaling Cascade in Normal Mice

**Figure 6.**
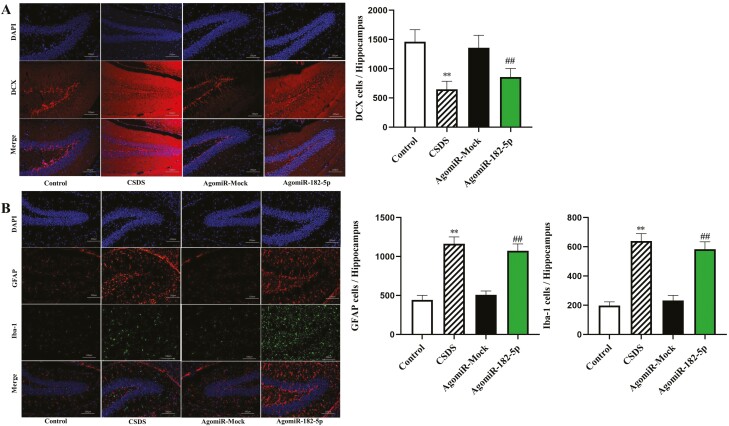
Overexpression of miR-182-5p downregulated the level of hippocampal neurogenesis in C57BL/6 J mice, brain sections (25 μm thick) were prepared 24 hours after AgomiR-182-5p treatment, and immunostaining for DCX, Iba-1, and GFAP were performed, along with quantification results of DCX, Iba-1, and GFAP (scale bar = 100 μm for representative images; n = 3). All data are means ± SEM. ^**^P < .01 compared with the control; ^##^*P < *.01 compared with the AgomiR-Mock. The comparisons were made by 1-way ANOVA with Tukey post hoc correction.

**Figure 7.**
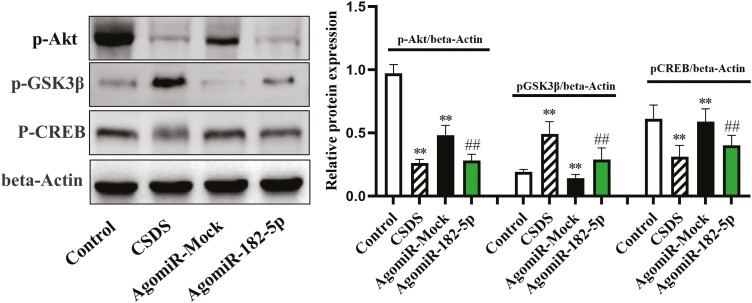
AgomiR-182-5p treatment decreased the hippocampal Akt/GSK3β/CREB signaling cascade in normal mice. Western-blot analysis showed effects of AgomiR-182-5p treatment on the expression of p-Akt, p-GSK3β, and p-CREB protein levels compared with AgomiR-Mock group. The results of analysis are means ± SEM, n = 3. ^**^P < .01 compared with the control group; ^##^*P < *.01 compared with the AgomiR-Mock group. The comparisons were made by 1-way ANOVA with Tukey post hoc correction.

Results from our immunofluorescence analysis showed that a decreased amount of DCX+ cells in the DG was observed in CSDS-induced mice, and the amount of DCX+ cells was robustly decreased in the DG of control mice receiving agomiR-182-5p administration [ANOVA: F_(3,16)_ = 51.483, *P* < .01] compared with that of controls receiving the infusion of AgomiR-Mock (Fig. 6A; n = 3). Similarly, compared with control mice, the amount of GFAP [ANOVA: F_(3,16)_ = 74.219, *P* < .01] and Iba-1 [ANOVA: F_(3,16)_ = 36.827, *P* < .01] cells were increased in the DG of mice subjected to agomiR-182-5p treatment (Fig. 6B; n = 3). Moreover, results from western-blot analysis showed that (Fig. 7; n = 3), in contrast to that observed in mock controls, the overexpression of miR-182-5p decreased protein levels of p-Akt and p-CREB and increased p-GSK3β expression in the hippocampus [p-Akt: F_(3,16)_ = 19.483, *P* < .01; p-GSK3β: F_(3,16)_ = 25.972, *P* < .01; p-CREB: F_(3,16)_ = 18.473, *P* < .01]. Again, findings were similar to the decreased p-Akt and p-CREB and increased p-GSK3β expression levels seen in mice following 10 days of CSDS exposure compared with that of controls receiving the infusion of 0.9% saline solution.

### Antidepressant Effects of AAV-siR-182-5p Need Involvement of Akt/GSK3β/CREB Signaling in CSDS-Exposed Mice

To demonstrate whether the hippocampal Akt/GSK3β/CREB signaling pathway is involved in the antidepressant effects of AAV-siR-182-5p, first of all, to eliminate the effects of 666-15 on the depressive behavior in CSDS-induced mice, we treated depressed mice with 666-15 and measured representations of behavior tests (supplementary Fig. 2A–D). Next, we co-injected CSDS-treated mice with AAV-siR-182-5p and 666-15; 666-15 is a specific inhibitor of CREB. AAV-siR-182-5p was stereotactic injected into the brain of mice and 666-15 was i.p. injected in a volume of 10 mg/kg (2 hours later) for 2 weeks, and then behavioral tests (FST, TST, SPT, and SIT) were conducted (supplementary Fig. 3A). With regarding to the behavioral measurement, the ANOVA showed that 666-15 significantly blocked the antidepressant effects of AAV-siR-182-5p against CSDS in the FST [AAV: F_(1,42)_ = 29.984, *P* < .01; AAV + 666-15: F_(3,42)_ = 25.528, *P* < .01; interaction: F_(3,42)_ =12.153, *P* < .01], TST [AAV: F_(1,42)_ = 31.635, *P* < .01; AAV + 666-15: F_(3,42)_ = 22.704, *P* < .01; interaction: F_(3,42)_ =18.535, *P* < .01], SPT [AAV: F_(1,42)_ = 18.335, *P* < .01; AAV + 666-15: F_(3,42)_ = 13.905, *P* < .01; interaction: F_(3,42)_ =10.827, *P* < .01], and SIT [AAV: F_(1,42)_ = 52.836, *P* < .01; AAV + 666-15: F_(3,42)_ = 42.572, *P* < .01; interaction: F_(3,42)_ = 31.774, *P* < .01] (supplementary Fig. 3B–E; n=10). Similarly, the ANOVA results from immunofluorescence analysis (supplementary Fig. 4A–B; n = 3) showed that 666-15 obviously abolished the effects of AAV-siR-182-5p on the increased hippocampal neurogenesis in the CSDS-stressed mice [DCX, AAV: F_(1,17)_ = 42.577; AAV + 666-15: F_(3,17)_ = 35.418; interaction: F_(3,17)_ = 28.439] [GFAP, AAV: F_(1,17)_ = 31.724; AAV + 666-15: F_(3,17)_ = 27.441; interaction: F_(3,17)_ = 18.636] [Iba-1, AAV: F_(1,17)_ = 28.559; AAV + 666-15: F_(3,17)_ = 19.927; interaction: F_(3,17)_ = 15.205].

## DISCUSSION

Of late, new evidence from increasing numbers of studies show that some sort of relationships exist between miRNA and depression in specific brain regions, and the potential molecular mechanisms involved have drawn researchers’ attention. Although the antidepressants are widely used in clinical practice, these medications face varying problems, such as poor compliance of patients, drug toxicity, high price, and so on (Dion-Albert et al., 2022). The results from our study have provided the first evidence, to our knowledge, directly confirming the importance of miR-182-5p in depression. First, we found that the level of miR-182-5p was significantly upregulated in the hippocampus (qRT-PCR method) within a CSDS mouse model of depression, in which the results were consistent with historical findings (Mizohata et al., 2021). Secondly, overexpression of miR-182-5p aggravated depression-like behaviors along with a progressive reduction in the number of DCX cells and the levels of Akt/GSK3β/CREB signal pathways as depression became worse. The loss of miR-182-5p, in comparison, effectively improved impaired neurogenesis, prevented the decreased expression of Akt/GSK3β/CREB signal pathway, and relieved the immobility time, amount of sugar water consumed, and social anxiety disorder in these CSDS mice. Finally, the use of 666-15 suggested that miR-182-5p was involved in the regulation of depression via the Akt/GSK3β/CREB biosynthesis.

The CSDS model of depression was successfully established in our study because the CSDS paradigm induced heterogeneous individual behavioral phenotypes among socially stressed mice (Golden et al., 2011). The findings were similar to those in previous studies that found CSDS persistently induced depression-like phenotypes characterized by social-avoidance behaviors, anhedonia, and despair, such as impaired social interaction in the SIT, lower sugar water consumption, and increased immobility time of mice (Shi et al., 2022). Furthermore, there are other comparatively effective models of depression, including the chronic unpredictable mild stress (CUMS) model and the chronic restraint stress model to simulate the depressive behaviors of rodents (Qian et al., 2021; Tang et al., 2021). It is possible that miR-182-5p may play critical roles in depression-like behaviors in CUMS and chronic restraint–induced mice models of depression.

In the current study, the effects of miR-182-5p on depression-like behaviors were observed in male mice. Recent research has shown that depression is more prevalent in females than in males (Bartova et al., 2021). It is known that sex hormones, menstrual cycle, and environmental factors may be involved in the greater female predisposition to develop depression (Baldini et al., 2021). However, the potential mechanisms of miR-182-5p on female mice have not been identified and deserve further study.

The symbol of depression is defective hippocampal neurogenesis (Feng et al., 2022). Neurogenesis defects in the hippocampus are associated with depression-related symptoms, such as hopelessness and helplessness (Anacker and Hen, 2017; Gomes-Leal, 2021). A growing body of research now shows that impaired neurogenesis alters a spectrum of physiological processes, such as the hypothalamic-pituitary-adrenal axis, inflammation, and neurotrophic factors (Cao et al., 2021; Lee et al., 2022), which are all critical to stress responses or depression. In addition, the effects of antidepressants were not sensitive to the defective neurogenesis; thus, it is difficult for drugs to play a useful role in depression. In practice, however, such additional underlying mechanisms of these pathophysiological processes in depression are not fully understood.

We focused on the neurotrophins because they were considered indicators involved in etiology and treatment of depression. We verified that overexpression of miR-182-5p not only significantly decreased expression levels of Akt/GSK3β/CREB signal pathway in the hippocampal area but also led to depression-like behaviors in CSDS mice. According to the neurotrophin hypothesis, lowered release of brain-derived neurotrophic factor (BDNF) caused neurogenesis damage in emotional memory-related brain regions, whereas an increase of BDNF-expression boosted neuronal plasticity (Wang et al., 2022). For example, Pingping-Tan et al. confirmed that impaired CREB-BDNF signaling led to the development of depression-like behaviors in the hippocampus of CUMS mice (Tan et al., 2022). In clinical studies, lower serum levels of BDNF in depressed patients were found compared with healthy individuals, and effective treatment promoted an increase in BDNF levels (Ding et al., 2022). In addition to BDNF, depression and neurodegeneration are associated with other decreased neurotrophic factors, like neurotrophic factor-α1 (NF-α1) and nerve growth factor (Aygun et al., 2022). For instance, Lan Xiao and Yoke Peng Loh proved NF-α1 functions in neuroprotection and alleviated depression in mice (Xiao and Loh, 2022). Therefore, we hypothesized that the potential mechanism of miR-182-5p involved in regulating depression might be mediated by targeting the expression level of BDNF, NF-α1, or nerve growth factor. More research is needed to confirm and explore the findings.

The usage of Akt/GSK3β/CREB signaling inhibitor (666-15) precluded this possibility, indicating that the Akt/GSK3β/CREB system was critical for the antidepressant effect of AAV-siR-182-5p on CSDS-induced mice. The efficacy and safety of the doses of 666-15 was based on previous study (Yan et al., 2021). Previous studies showed that CREB acted as a major transcription factor in brain development and neurogenesis, CREB was activated in a phosphorylated form, and multiple protein kinases phosphorylated this transcription factor and converted CREB to its active form (Keshavarzi et al., 2019). Akt in brain cells could activate GSK3, which was involved in neurodegeneration and inhibited protecting cells from neurodegenerative effects of GSK3 (Chen et al., 2004). For example, the studies of Meihua-Wang et al. showed that natural product geniposide ameliorated depression-like behaviors via the phosphatidylinositol 3-kinase (PI3K)/Akt/GSK3β axis in CUMS-induced mice (Wang et al., 2021). A major cause of depression is considered as a disruption of homeostasis in neurological function in the central nervous system (Duman and Aghajanian, 2012). MiRNAs have been shown to contribute to the disruption via regulating gene expression related to neurogenesis, synaptic plasticity, and neurotransmission (Murphy and Singewald, 2019). It had been reported that miR-182 was upregulated in peripheral blood of patients with depression and CUMS-induced rats (Li et al., 2013; Li et al., 2016). Protein kinase B, commonly known as Akt, was abundantly expressed in mammals. Akt signaling affected brain production and activity for a number of neurological diseases by being involved in neuronal survival and growth (Manning and Toker, 2017). Multiple studies have shown miR-182-5p participated in various illnesses via the activation or suppression of Akt signal pathway (Huang et al., 2018; Huang et al., 2018; Yao and Yan, 2020), demonstrating that miR-182-5p had some kind of connection to the Akt signaling. On the other hand, some molecular studies demonstrated that effects of these AMP kinases were modulated by CREB/BDNF and Akt/GSK3 signaling pathways, which played important roles in depression (Fryer et al., 2002; Lu and Xu, 2006). So, we assumed miR-182-5p might mediate inhibitory effects of the Akt/GSK3β/CREB pathway in the hippocampus of mice with depressive-like behaviors.

The remarkable thing is that miR-182-5p is more likely to be involved in regulating depression via other signaling cascades, which will be examined in the future.

There are also some limitations in this study that should be addressed in future investigations. First, we only used young mice (7–8 weeks old) in our study. Depression in elderly people is a major public health concern, and many elderly patients have unsatisfactory responses to antidepressants (Tham et al., 2016), including problematic side effects and the risk of drug interactions. Secondly, although the miR-182-5p was a focal point of research in hippocampus, miR-182-5p may also show differential expression in the ventraltegmental area and medial prefrontal cortex. Third, because chronic stress leads to hyperactivity of the hypothalamic-pituitary-adrenal axis, the level of plasma corticosterone in mice needs to be evaluated in the future.

In summary, our findings reveal a crucial role for miR-182-5p in depressive symptoms of mice through its capacity to regulate neurogenesis and specific neurotrophic factors. We believe targeting miRNAs may be therapeutic strategies for the development of new antidepressant drugs in the treatment of depression. Meanwhile, we look forward to future progress of miR-182-5p–based therapies in depression in the next few years.

## CONCLUSION

Overall, the expression level of miR-182-5p was upregulated in the hippocampus of CSDS-induced mice, and administration of AAV-siR-182-5p ameliorated depression-like behaviors and defective neurogenesis in CSDS-induced mice, which seemed to be facilitated via activation of the hippocampal Akt/GSK3β/CREB signaling pathway.

## Supplementary Material

pyad064_suppl_Supplementary_Figure_S1

pyad064_suppl_Supplementary_Figure_S2

pyad064_suppl_Supplementary_Figure_S3

pyad064_suppl_Supplementary_Figure_S4

pyad064_suppl_Supplementary_Material

## Data Availability

The data underlying this article will be shared on reasonable request to the corresponding author.
