# Supplementary figures and images for: MiR-182-5p: A Novel Biomarker in the Treatment of Depression in CSDS-Induced Mice
Source: Int J Neuropsychopharmacol. 2023 Dec 1;27(1):pyad064. doi: 10.1093/ijnp/pyad064 (PMC10799762; doi:10.1093/ijnp/pyad064)

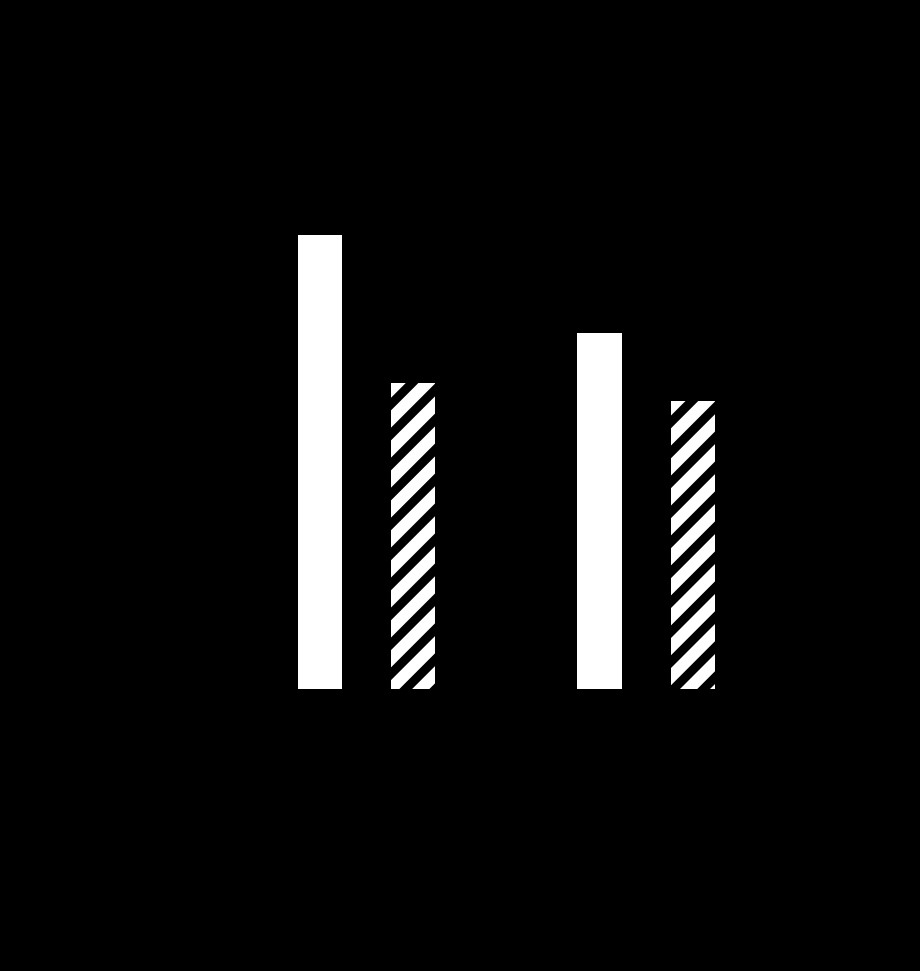

Supplement: pyad064_suppl_Supplementary_Figure_S1 [file pyad064_suppl_supplementary_figure_s1.jpeg]

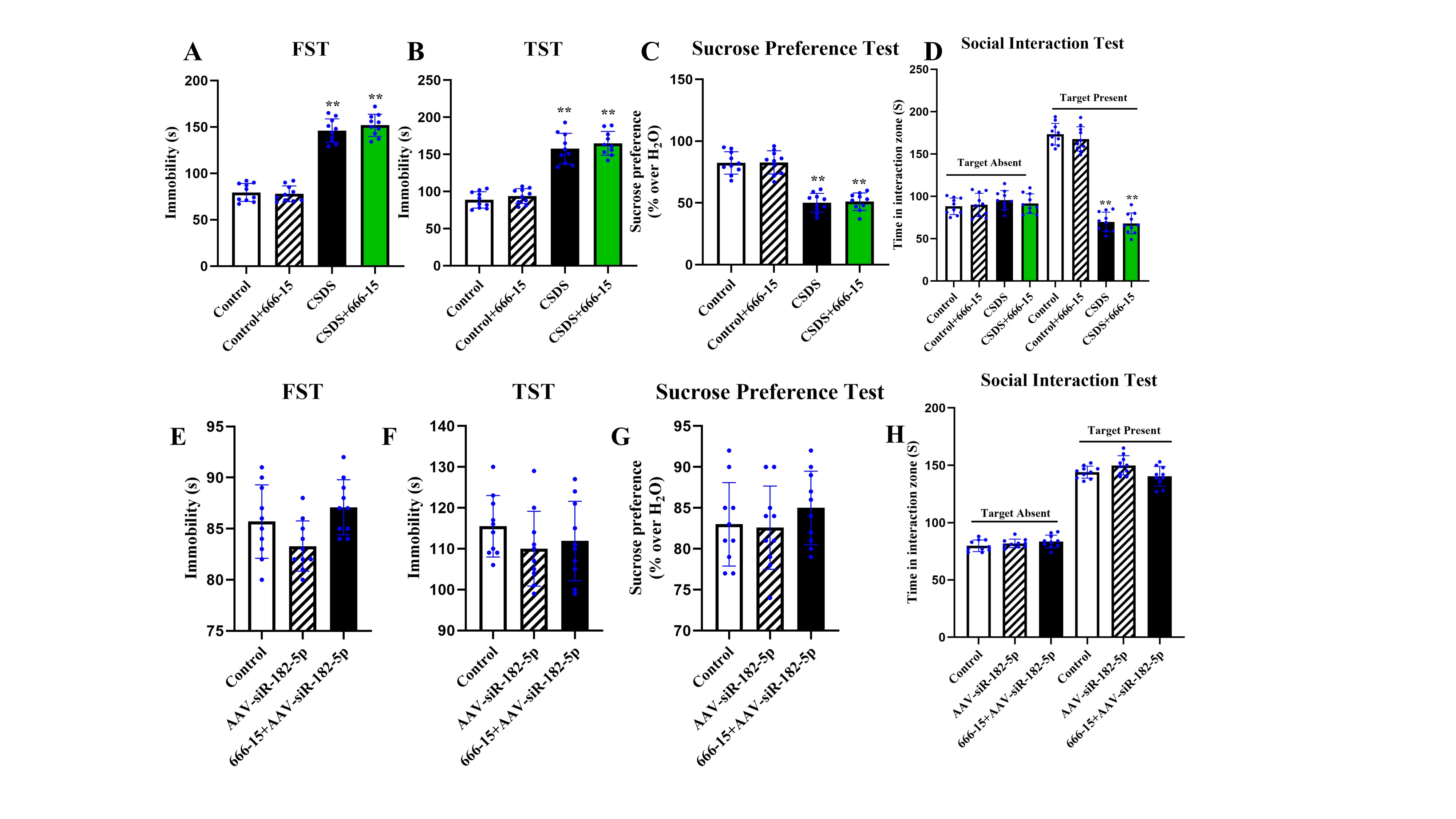

Supplement: pyad064_suppl_Supplementary_Figure_S2 [file pyad064_suppl_supplementary_figure_s2.jpeg]

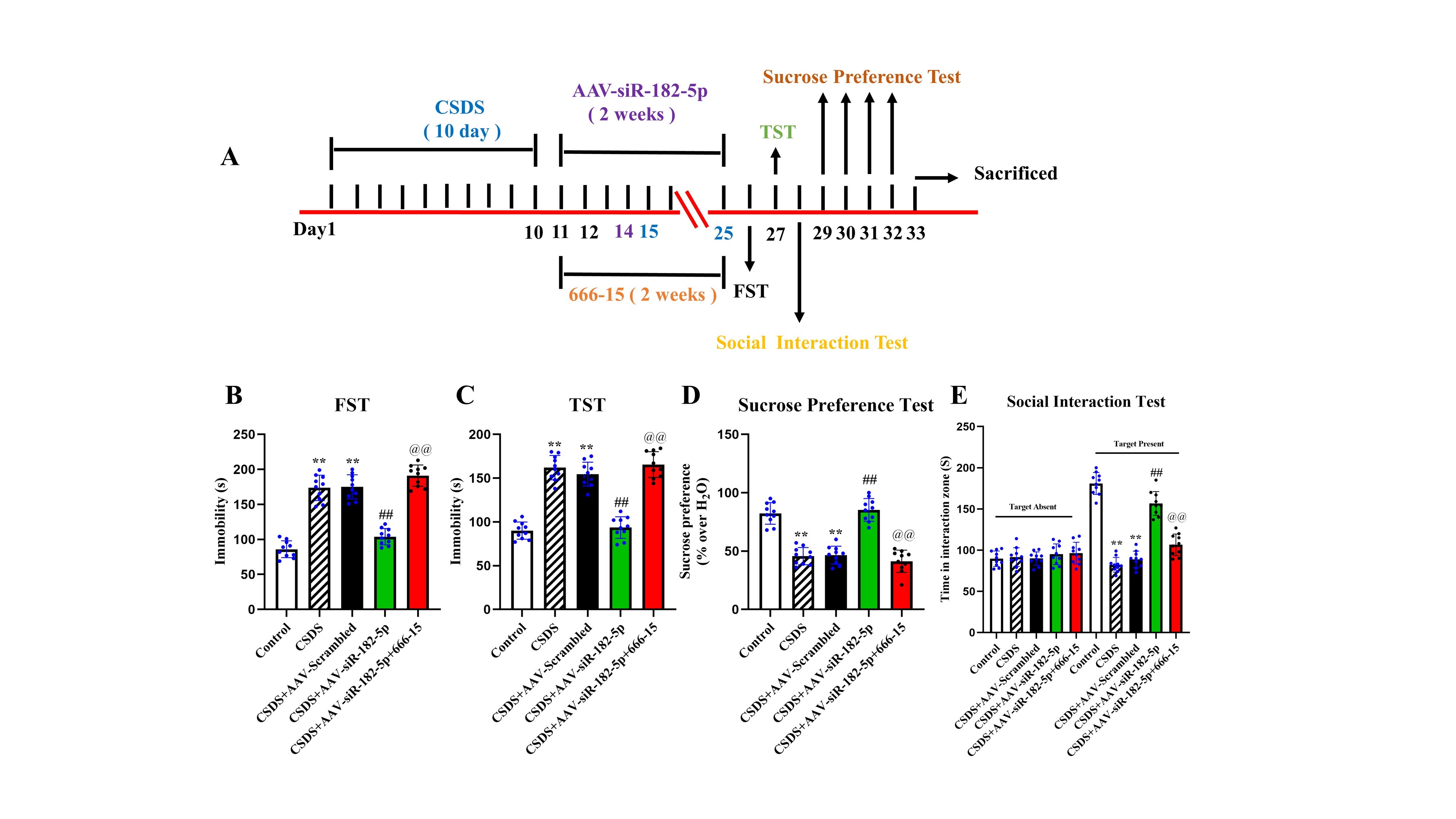

Supplement: pyad064_suppl_Supplementary_Figure_S3 [file pyad064_suppl_supplementary_figure_s3.jpeg]

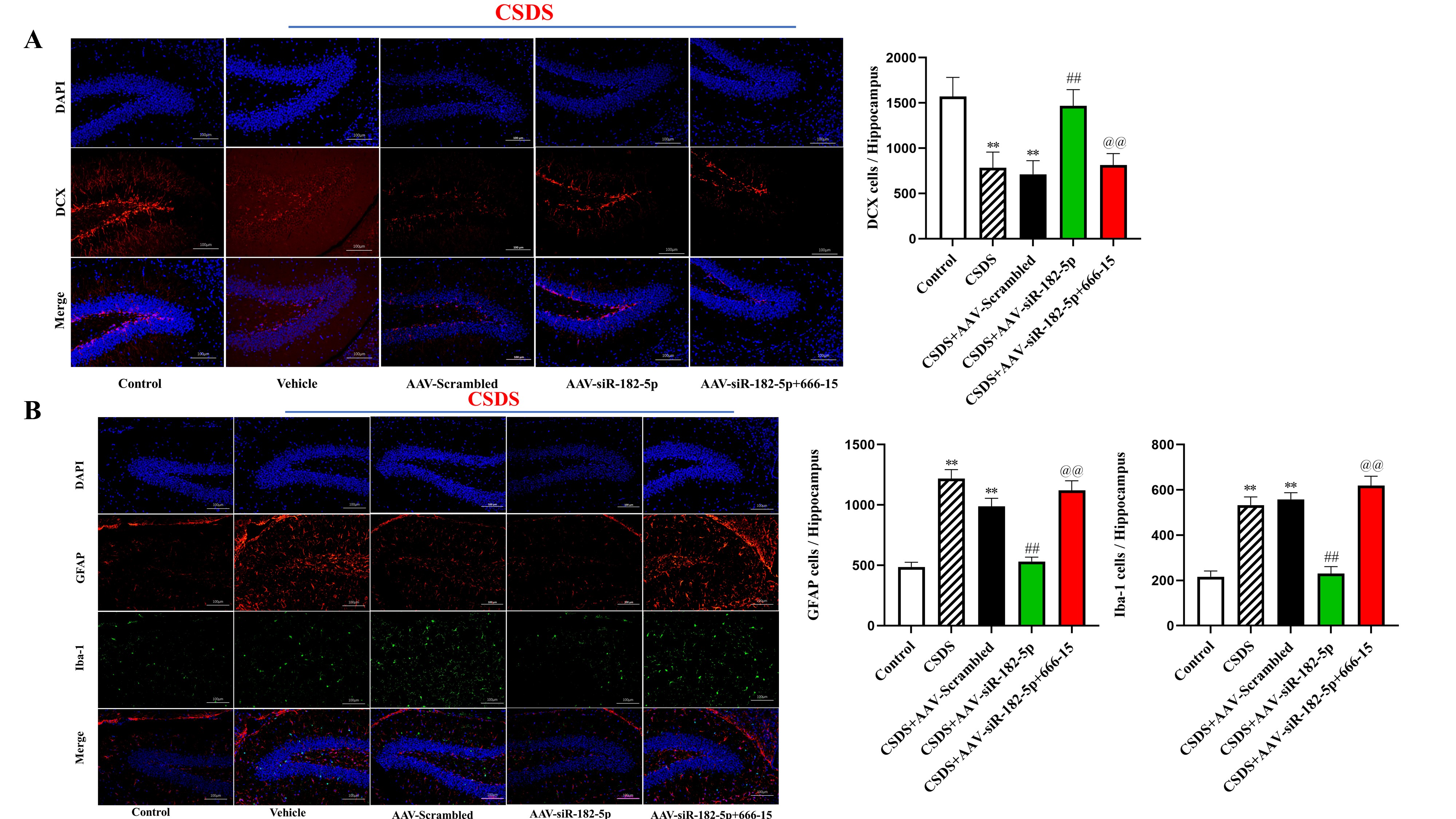

Supplement: pyad064_suppl_Supplementary_Figure_S4 [file pyad064_suppl_supplementary_figure_s4.jpeg]
