# Supplementary material for: MiR-182-5p: A Novel Biomarker in the Treatment of Depression in CSDS-Induced Mice
Source: Int J Neuropsychopharmacol. 2023 Dec 1;27(1):pyad064. doi: 10.1093/ijnp/pyad064 (PMC10799762; doi:10.1093/ijnp/pyad064)
Supplement: pyad064_suppl_Supplementary_Material [file pyad064_suppl_supplementary_material.docx]

**Figs. S1**

RNA-protein immunoprecipitation (RIP)

RIP assays were performed by the Magna RIP Kit (Millipore, Billerica, MA) according to the manufacturer’s instructions. Hippocampal tissues of CSDS-induced mice were incubated with RIP lysis buffer containing RNase inhibitor and protease inhibitor. Then, lysates were immunoprecipitated with anti-IgG or anti-AGO2-conjugated magnetic beads at 4°C overnight (Normal rabbit IgG was used as the negative control). Finally,

the immunoprecipitated RNA complex were then purified and quantified by qRT-PCR.

AKT was a target of miR-182-5p

From previous studies, it's known that the molecular mechanism by which miR-182-5p contributes to some illnesses via activating the AKT/FOXO3a signaling pathway(Cao MQ et al., 2018;Xu X et al., 2014). In order to further prove the reliability of combination between miR-182-5p and AKT, a RIP assay was designed especially. The results showed that the levels of miR-182-5p and AKT were enriched in AGO2 (Figs.S2) in the hippocampus of CSDS-induced mice.


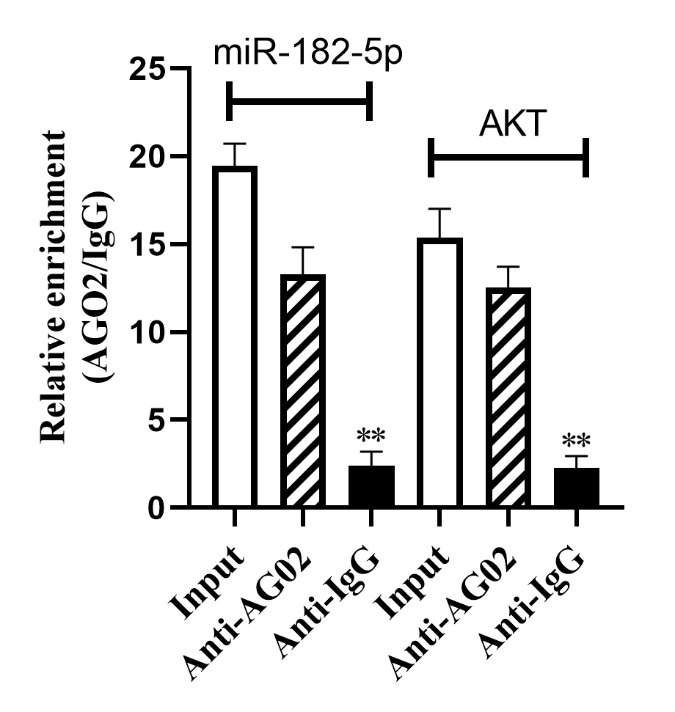


**Figs. S1.** AKT was more likely a target of miR-182-5p. RIP assay was used to evaluate the interaction between AKT and miR-182-5p (n = 3). Data are shown as mean ± SEM. ^**^*P* < 0.01 when compared to the Anti-AGO2.


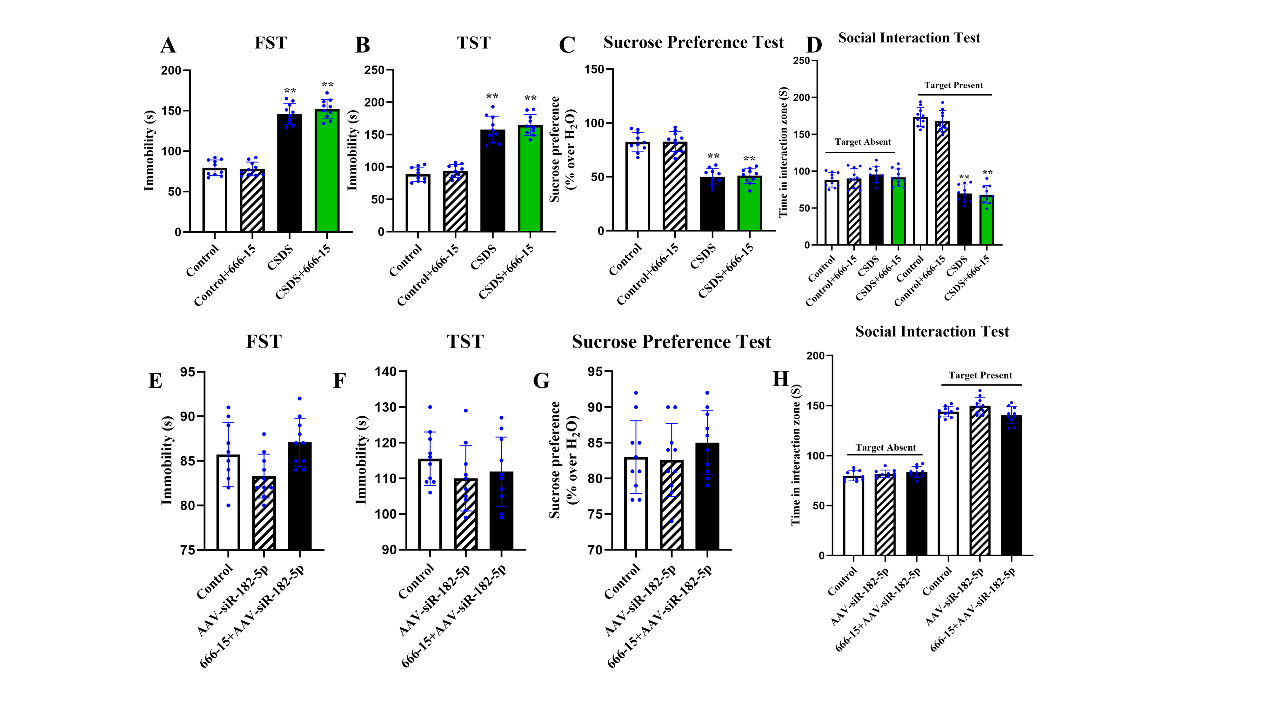
**Figs. S2**

**Figs. S2.** Both 666-15 and AAV-siR-182-5p had no significant influence on behaviors of normal mice and depressive-like behaviors of CSDS-induced mice. (A-D) CSDS induced notable depressive-like actions of C57BL/6J mice in the FST, TST, SPT and SIT, however, there was no significant difference in behaviors of mice treated with 666-15 as compared with normal or CSDS-induced mice (n = 10). In addition, behavior test showed no significant difference between normal mice and mice treated with 666-15 and AAV-siR-182-5p. Data are shown as mean ± SEM. ^**^*P* < 0.01 when compared to the Control.


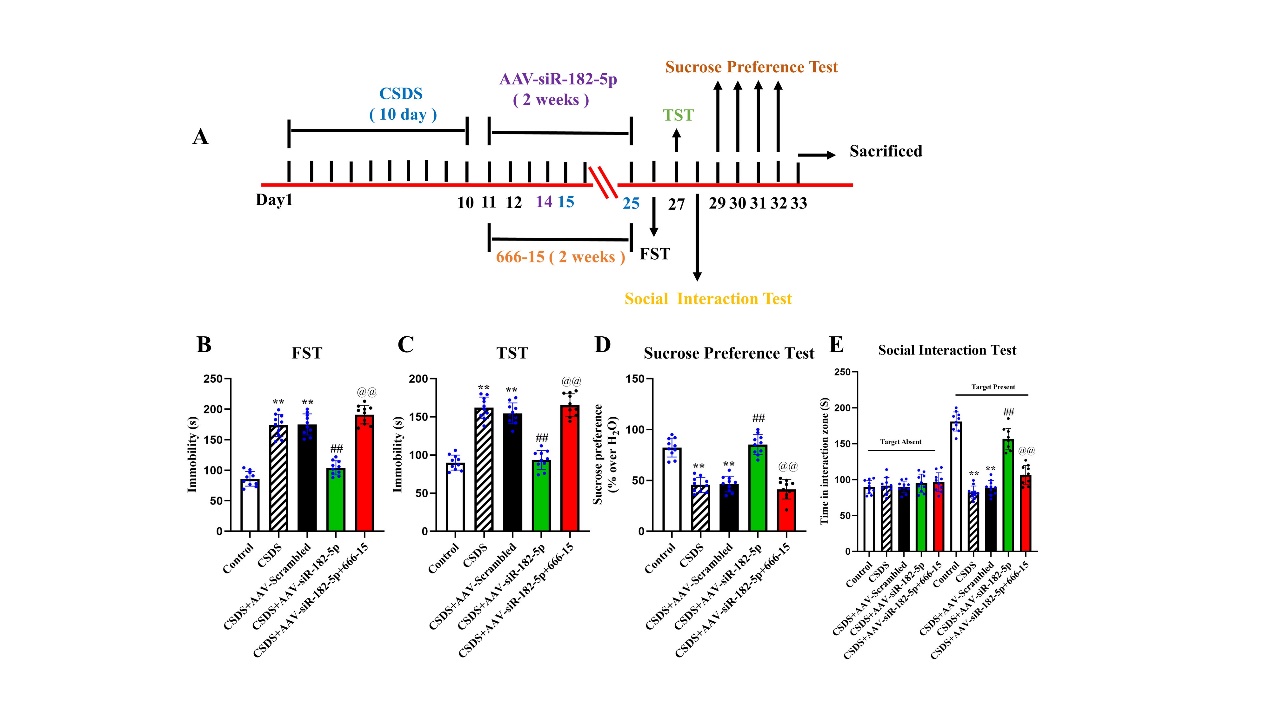
**Figs. S3**

**Fig. S3.** Blockage of Akt/GSK3β/CREB signaling by 666-15 obviously restrained the antidepressant activity of AAV-siR-182-5p. (A) Schematic timeline of CSDS, viral and 666-15 injection, and behavioral tests. (B-E) Immobility time in the FST and TST, the intake of sucrose (in percentage) in the SPT andthe interaction time in the SIT (n = 10). The results of analysis were expressed as the means ± SEM. ^**^*P* < 0.01 when compared to Control; ^##^*P <* 0.01 when compared to CSDS; ^@@^*P <* 0.01 when compared to CSDS + AAV-siR-182-5p. Two-way ANOVA was used for multiple comparisons involving > 2 groups.

**Figs. S4**


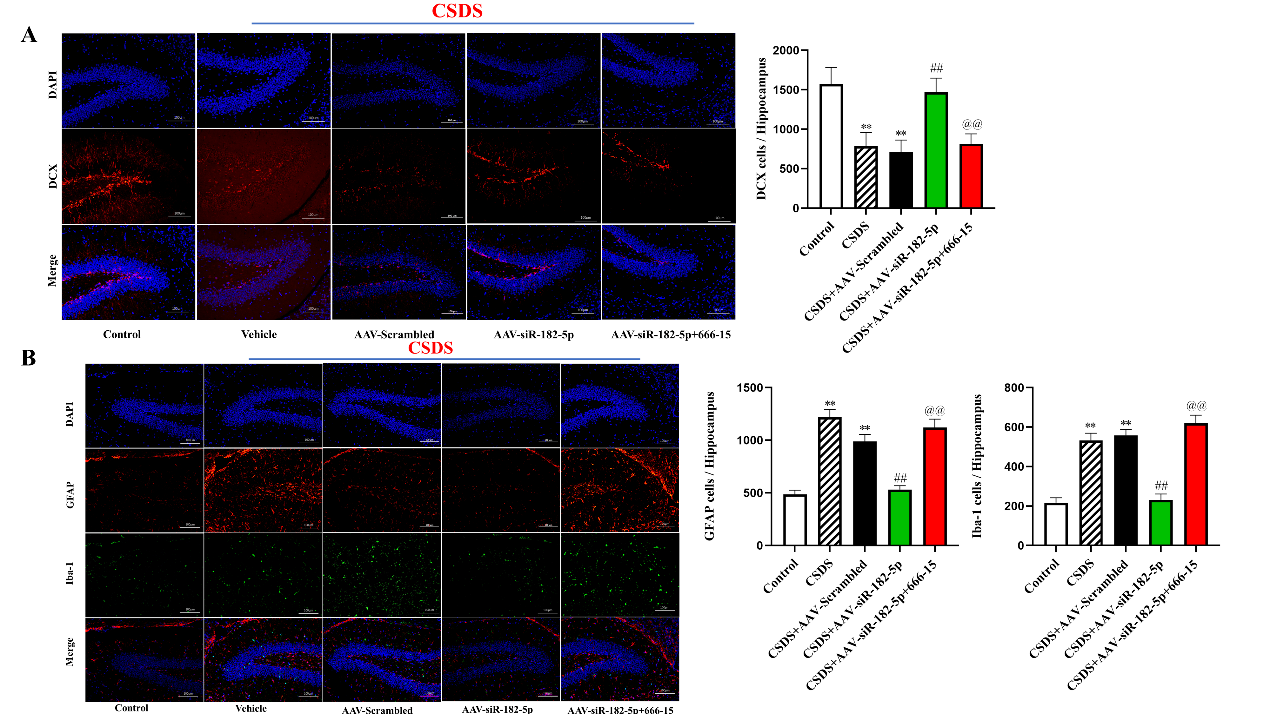


**Fig. S4.** Impact of pretreatment with 666-15 on the number of DCX (A), GFAP and Iba-1 cells (B). Statistics analyses showed that the increase of DCX neurons in the hippocampus of the CSDS-treated mice were significantly blocked by 666-15 in AAV-siR-182-5p group, and decreased numbers of GFAP and Iba-1 cells were also prevented by 666-15 (The scale bar is 100 μm for the representative images; n = 3). All data were shown as the means ± SEM. ^**^*P* < 0.01 when compared to the Control; ^##^*P <* 0.01 when compared to the CSDS; ^@@^*P <* 0.01 when compared to CSDS + AAV-siR-182-5p. Two-way ANOVA was used for multiple comparisons involving > 2 groups.

Cao MQ, You AB, Zhu XD, Zhang W, Zhang YY, Zhang SZ, Zhang KW, Cai H, et al. (2018), miR-182-5p promotes hepatocellular carcinoma progression by repressing FOXO3a. J Hematol Oncol 11:12.

Xu X, Wu J, Li S, Hu Z, Xu X, Zhu Y, Liang Z, Wang X, et al. (2014), Downregulation of microRNA-182-5p contributes to renal cell carcinoma proliferation via activating the AKT/FOXO3a signaling pathway. Mol Cancer 13:109.
